# Does amyloid deposition produce a specific atrophic signature in cognitively normal subjects?^☆^

**DOI:** 10.1016/j.nicl.2013.01.006

**Published:** 2013-01-24

**Authors:** Jennifer L. Whitwell, Nirubol Tosakulwong, Stephen D. Weigand, Matthew L. Senjem, Val J. Lowe, Jeffrey L. Gunter, Bradley F. Boeve, David S. Knopman, Bradford C. Dickerson, Ronald C. Petersen, Clifford R. Jack

**Affiliations:** a Department of Radiology, Mayo Clinic, Rochester, MN, USA; b Department of Health Sciences Research (Biostatistics), Mayo Clinic, Rochester, MN, USA; c Department of Information Technology, Mayo Clinic, Rochester, MN, USA; d Department of Neurology, Mayo Clinic, Rochester, MN, USA; e Department of Neurology, Massachusetts General Hospital and Harvard Medical School, Boston, MA, USA; f Department of Epidemiology, Mayo Clinic, Rochester, MN, USA

**Keywords:** Amyloid, Preclinical, Alzheimer's disease, Freesurfer, Voxel-based morphometry, Cognitively normal

## Abstract

The objective of our study was to evaluate whether cognitively normal (CN) elderly participants showing elevated cortical beta-amyloid (Aβ) deposition have a consistent neuroanatomical signature of brain atrophy that may characterize preclinical Alzheimer's disease (AD). 115 CN participants who were Aβ-positive (CN +) by amyloid PET imaging; 115 CN participants who were Aβ-negative (CN −); and 88 Aβ-positive mild cognitive impairment or AD participants (MCI/AD +) were identified. Cortical thickness (FreeSurfer) and gray matter volume (SPM5) were measured for 28 regions-of-interest (ROIs) across the brain and compared across groups. ROIs that best discriminated CN − from CN + differed for FreeSurfer cortical thickness and SPM5 gray matter volume. Group-wise discrimination was poor with a high degree of uncertainty in terms of the rank ordering of ROIs. In contrast, both techniques showed strong and consistent findings comparing MCI/AD + to both CN − and CN + groups, with entorhinal cortex, middle and inferior temporal lobe, inferior parietal lobe, and hippocampus providing the best discrimination for both techniques. Concordance across techniques was higher for the CN − and CN + versus MCI/AD + comparisons, compared to the CN − versus CN + comparison. The weak and inconsistent nature of the findings across technique in this study cast doubt on the existence of a reliable neuroanatomical signature of preclinical AD in elderly PiB-positive CN participants.

## Highlights

► We measured atrophy in cognitively normal subjects with amyloid deposition (CN +). ► Findings in CN + subjects were weak and disconcordant across Freesurfer and SPM5. ► Concordance across techniques was higher when assessing Alzheimer disease subjects. ► Evidence for a neuroanatomical signature of preclinical AD in CN + subjects is weak.

## 1 Introduction

Alzheimer's disease (AD) is characterized by the deposition of beta-amyloid (Aβ) senile plaques and tau-positive neurofibrillary tangles (NFTs) in the brain (Braak and Braak, 1991). It is associated with characteristic patterns of atrophy preferentially involving medial and lateral temporal lobes, and parietal and frontal association cortices. In concordance with the topographic progression of NFTs (Braak and Braak, 1991) at autopsy, early atrophic changes have been observed in entorhinal cortex and hippocampus, before spreading throughout temporal and parietal lobes, and then later affecting frontal lobes (Whitwell et al., 2007). Several studies assessing cognitively normal (CN) subjects who show positive Aβ PET scanning, and hence presumed preclinical AD (Sperling et al., 2011), have observed atrophy in these subjects (Dickerson et al., 2009a, 2011; Mormino et al., 2009; Storandt et al., 2009; Becker et al., 2011; Bourgeat et al., 2010; Chetelat et al., 2010; Oh et al., 2010), with some reporting a direct link between the presence of Aβ and atrophy in the frontal and parietal cortices (Becker et al., 2011; Chetelat et al., 2010; Oh et al., 2010). One of these studies even suggested that these Aβ-related neocortical neurodegenerative changes may occur prior to neurodegeneration in the medial temporal lobe (Becker et al., 2011). This challenges the topographic pattern of progression in AD, and has important consequences for the current model of pathological sequences in AD which proposes that Aβ deposition precedes tau deposition and that tau deposition leads to neurodegeneration (Jack et al., 2010).

The aim of this study was therefore to evaluate evidence for a neuroanatomical signature of Aβ deposition in a cohort of CN elderly who had undergone Aβ PET scanning with Pittsburgh Compound B (PiB). Since the majority of studies that associated atrophy with Aβ deposition utilized FreeSurfer software and those that associated atrophy with tau deposition utilized Statistical Parametric Mapping (SPM) software, we utilized both techniques in the current study to determine whether differences in the literature could be driven in part by variability due to technique and whether a consistent neuroanatomic signature of Aβ deposition could be identified across technique.

## 2 Design and methods

### 2.1 Participant selection

We identified all CN participants from the Mayo Clinic Alzheimer's Disease Research Center (ADRC) or the Mayo Clinic Study of Aging (MCSA) with PiB-PET and MRI (n = 330). These participants were divided into PiB-negative (CN −) (n = 215) or PiB-positive (CN +) (n = 115) based on methods described below. In order to control for possible confounding due to age, sex, and education, we compared all 115 CN + participants to a matched set of 115 CN − participants (matching using propensity scores; D'Agostino, 1998). In addition to assessing differences between CN − and CN + participants, we wanted to assess atrophy in a group with a known and homogeneous neuroanatomical signature. Therefore, we identified a group of ADRC or MCSA patients with clinical diagnoses of amnestic MCI or AD who were PiB-positive (MCI/AD +) (n = 88). Demographics of the groups are shown in Table 1).

The ADRC recruits individuals seeking medical care at the Mayo Clinic and the MCSA is an epidemiologic study of normal aging and MCI in 70–90 year olds in Olmsted County, Minnesota (Roberts et al., 2008); both are prospective longitudinal studies that perform similar clinical and cognitive assessments and identical imaging protocols. In all cases the most recent MRI was used for analysis and the PiB scan was performed within 5 months of the MRI (median interval = 20 days).

Diagnoses were made on a clinical basis at consensus conferences including neurologists, neuropsychologists, a neuropsychiatrist and study coordinators (Roberts et al., 2008; Petersen et al., 2010). Participants were characterized as CN if they were judged to be cognitively normal and their age-adjusted neuropsychological test scores were consistent with normative data developed independently in this community (Ivnik et al., 1992). The presence/absence of subjective memory complaints in the CN subjects was measured based on the first 5 questions from the Blessed Memory Test, as previously detailed (Mielke et al., 2012). The diagnosis of amnestic MCI was made on clinical grounds, based on the presence of memory complaints and impairment on psychometric testing, without the use of rigid cutoffs on psychometric scores (Petersen, 2004). Alzheimer's disease was diagnosed using established criteria (McKhann et al., 1984). Informed consent was obtained from all participants or proxies for participation in the studies, which were approved by the Mayo Institutional Review Board.

### 2.2 PiB PET processing and classification

PET images were acquired using a PET/CT scanner (DRX; GE Healthcare) operating in 3-dimensional mode. Participants were injected with PiB (average, 596 MBq; range, 292–729 MBq) and after a 40 min uptake period a 20 min PiB scan was obtained consisting of four 5-minute dynamic frames (256 m FOV; pixel size = 1.0 mm; slice thickness = 3.3 mm).

PiB processing was performed using an automated image processing pipeline previously described in detail (Jack et al., 2008a). A global cortical PiB retention summary was formed by calculating the median uptake value among all voxels in prefrontal, orbitofrontal, parietal, temporal, anterior cingulate, and posterior cingulate/precuneus regions and dividing this by the median uptake in the cerebellar gray matter. Participants were classified as PiB-positive or negative using a global cortical-to-cerebellar ratio cut-point of 1.5 (Jack et al., 2008a).

### 2.3 MRI processing

All participants were imaged on a 3.0 T GE scanner with a 3D magnetization prepared rapid acquisition gradient echo (MPRAGE) sequence (Jack et al., 2008b). Parameters were: sagittal plane, TR/TE/TI, 2300/3/900 ms; flip angle 8°, 26 cm field of view (FOV); 256 × 256 in-plane matrix with a phase FOV of 0.94 and slice thickness of 1.2 mm. All images were corrected for gradient non-linearity and intensity inhomogeneity.

We used two common but substantively different techniques in our analysis: cortical thickness estimates from FreeSurfer and volume estimates from SPM5. FreeSurfer calculates cortical thickness using an estimate of the width of the cortical gray matter (Fischl and Dale, 2000), while SPM5 calculates volume based on segmenting tissue into gray and white matter using prior probability maps (Ashburner and Friston, 2000). While these techniques assess two different metrics, both are markers of cortical neurodegenerative atrophy. Assessing both techniques allows us to first determine whether we can replicate previous studies that have used these techniques, and secondly to assess whether the two techniques provide consistent results which would add strength to the proposition that Aβ is associated with a specific pattern of neurodegeneration in preclinical AD. FreeSurfer cortical thickness and SPM5 gray matter volume were used to assess ROI-derived and whole-cortex/whole-brain patterns of atrophy in CN + compared to CN −, MCI/AD + compared to CN − and MCI/AD + compared to CN +.

#### 2.3.1 FreeSurfer

FreeSurfer software version 4.5.0 (Dale et al., 1999; Fischl et al., 1999) (http://surfer.nmr.mgh.harvard.edu/) was utilized. Briefly, all images underwent skull-stripping and transformations into Talaraich space, followed by subcortical segmentation, identification of the gray/white boundary, automated topology correction, and surface deformation (Dale et al., 1999; Fischl and Dale, 2000). The resulting cortical models were registered to a spherical atlas (Fischl et al., 1999). The cerebral cortex was parcellated into regions based on gyral and sulcal structure. The atlas used for parcellation was created using 40 normal and AD participants. FreeSurfer outputted volume of hippocampus and amygdala, and cortical thickness for all other ROIs. Vertex-level comparisons of cortical thickness were also performed across groups using two sample t-tests after the spherical data was smoothed at 10 mm full-width at half-maximum (FWHM). A very lenient Statistical threshold of p < 0.005 uncorrected for multiple comparisons was applied.

#### 2.3.2 SPM5

Using SPM5 (http://fil.ion.ucl.ac.uk), a customized template was created using 200 CN and 200 AD participants (Vemuri et al., 2008). All 400 scans were normalized to the MNI template and segmented using unified segmentation into gray matter (GM), white matter (WM) and CSF. Average probability maps of GM, WM, and CSF were created and smoothed using 8 mm FWHM smoothing kernel to create customized tissue probability maps. Images from all subjects in the study were then normalized and segmented using unified segmentation and the customized tissue probability maps. Atlas-based parcellation was performed using the automated anatomic labeling (AAL) atlas (Tzourio-Mazoyer et al., 2002). The AAL atlas was normalized to the customized template, and each ROI was edited on the template in order to improve the accuracy of the atlas. In addition, the AAL parahippocampal gyrus ROI was split into parahippocampal gyrus and entorhinal cortex in order to better match the FreeSurfer atlas regions. The atlas was then warped into participant native space using the inverse transformations from above. Gray matter segmentations for each participant were parcellated into ROIs using the native-space atlas and gray matter volume estimated for each ROI. Voxel-level comparisons were performed using voxel-based morphometry (VBM) (Ashburner and Friston, 2000). The template-space segmented gray matter images were modulated for both the linear and non-linear components and smoothed at 8 mm FWHM, and comparisons were performed across groups using two sample t-tests, including TIV as a covariate. A very lenient statistical threshold of p < 0.005 uncorrected for multiple comparisons was applied.

**Supplemental Fig. 1:**
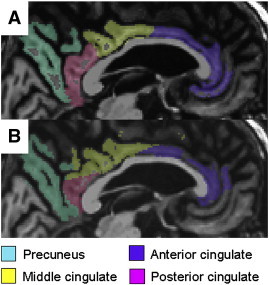
Gray matter segmentations of the cingulate gyrus and precuneus using FreeSurfer (A) and SPM5 with the AAL atlas (B) on the same control subject.

The atlases utilized in FreeSurfer and SPM5 differ, with FreeSurfer parcellating the cortex into 80 ROIs (Desikan et al., 2006), and the AAL atlas parcellating the cortex into 120 ROIs (Tzourio-Mazoyer et al., 2002). Therefore, we created a common set of 28 new ROIs that covered frontal, temporal, parietal, occipital lobes and insula. Supplemental Table 1 details how the individual ROIs from both techniques were utilized to create the 28 new ROIs. Volumes were summed and cortical thickness measures averaged to create the new ROIs. Supplemental Fig. 1 compares the segmentations from both techniques for some example medial regions. Left and right hemisphere volumes were summed and we calculated an average of left and right cortical thickness values weighted according to the surface area of the ROIs using the following formula: ((left surface area ∗ left thickness + right surface area ∗ right thickness) / left + right surface area). All ROI volumes from FreeSurfer were divided by the FreeSurfer total intracranial volume (TIV). Volumes from SPM5 were divided by a TIV calculated in SPM5 by propagating a template-drawn TIV mask to the subject space, and then performing an erosion step to remove border voxels. The FreeSurfer and SPM5 TIV measurements were highly correlated (R = 0.9). Since the thickness of most cortical regions does not relate to head size, thickness measures were not adjusted for TIV.

The atlases utilized in FreeSurfer and SPM5 differ, with FreeSurfer parcellating the cortex into 80 ROIs (Desikan et al., 2006), and the AAL atlas parcellating the cortex into 120 ROIs (Tzourio-Mazoyer et al., 2002). Therefore, we created a common set of 28 new ROIs that covered frontal, temporal, parietal, occipital lobes and insula. Supplemental Table 1 details how the individual ROIs from both techniques were utilized to create the 28 new ROIs. Volumes were summed and cortical thickness measures averaged to create the new ROIs. Supplemental Fig. 1 compares the segmentations from both techniques for some example medial regions. Left and right hemisphere volumes were summed and we calculated an average of left and right cortical thickness values weighted according to the surface area of the ROIs using the following formula: ((left surface area ∗ left thickness + right surface area ∗ right thickness) / left + right surface area). All ROI volumes from FreeSurfer were divided by the FreeSurfer total intracranial volume (TIV). Volumes from SPM5 were divided by a TIV calculated in SPM5 by propagating a template-drawn TIV mask to the subject space, and then performing an erosion step to remove border voxels. The FreeSurfer and SPM5 TIV measurements were highly correlated (R = 0.9). Since the thickness of most cortical regions does not relate to head size, thickness measures were not adjusted for TIV.

### 2.4 Statistical analysis

ROI-level analyses were based on two-sample Wilcoxon/Mann–Whitney tests and summarized by the area under the receiver operating characteristic curve (AUROC) which can be calculated directly from this test and interpreted as a measure of effect size independent of the underlying scales of measurement (Newcombe, 2006). For CN participants, the AUROC can be interpreted as the proportion of times that an arbitrary CN − participant would have greater cortical thickness (or volume) in an ROI compared to an arbitrary CN + participant. Values near 0.50 indicate poor discrimination, while a value near, for example, 0.75 would indicate that three-quarters of the time a CN + participant would have reduced gray matter compared to a CN − participant.

We present ROI-level AUROCs ranked from the highest/best (rank = 1) to the lowest/worst (rank = 28). Because of the underlying uncertainty in the rank of a given ROI due to sampling variability, we include 95% bootstrap confidence intervals for the rank of an ROI based on B = 1000 replicates. In addition, we calculated concordance correlation coefficients (CCC) to measure the agreement in terms of ROI rank across the techniques.

In order to assess the influence of PiB cut-point the analyses were repeated utilizing a PiB cut-point of 1.4 and removing patients with intermediate PiB values between 1.3 and 1.5. The analysis was also repeated using FreeSurfer volume measurements rather than thickness in order to account for any confounds related to these different measurements.

We used Spearman nonparametric correlations to examine the relationship between volume or thickness obtained from FreeSurfer and volume obtained from SPM5.

## 3 Results

### 3.1 ROI-level analysis

**Fig. 1:**
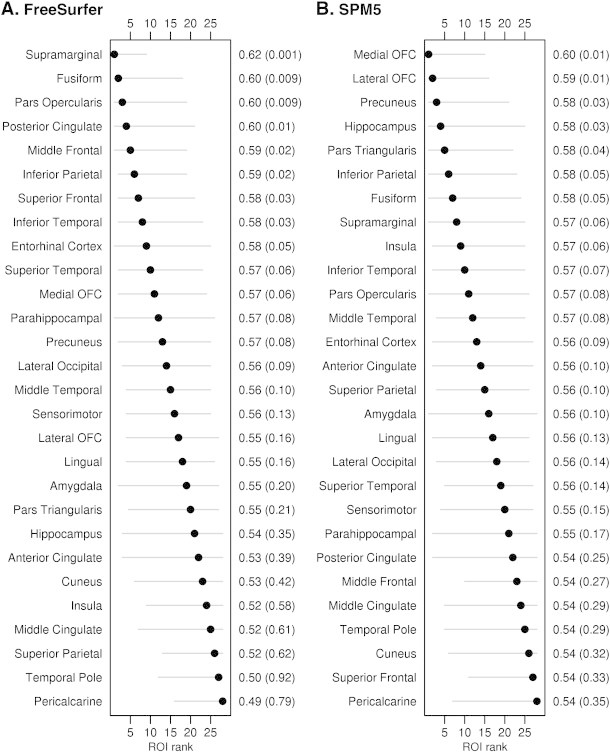
Forest plots illustrating ROI discrimination of CN − and CN + participants for FreeSurfer and SPM5. Regions-of-interest are ranked from best (rank of 1) to worst (rank of 28) according to AUROC and 95% bootstrap confidence intervals are shown. The second y axis shows the AUROC values and p values.

Both FreeSurfer and SPM5 identified ROIs that were significantly different between CN − and CN +, with discrimination for the top ROIs showing AUROCs in the 0.60 range (Fig. 1). For FreeSurfer cortical thickness, the five ROIs that showed best discrimination were supramarginal gyrus, fusiform gyrus, pars opercularis, posterior cingulate, and middle frontal gyrus. For SPM5 gray matter volume, the five best ROIs were medial orbitofrontal cortex, lateral orbitofrontal cortex, precuneus, hippocampus, and pars triangularis. In addition to the disagreement in terms of which ROIs provided the best discrimination, wide confidence intervals of equivalent magnitudes were observed for both techniques indicating there is little certainty within a technique as to the top ROIs.

**Fig. 2:**
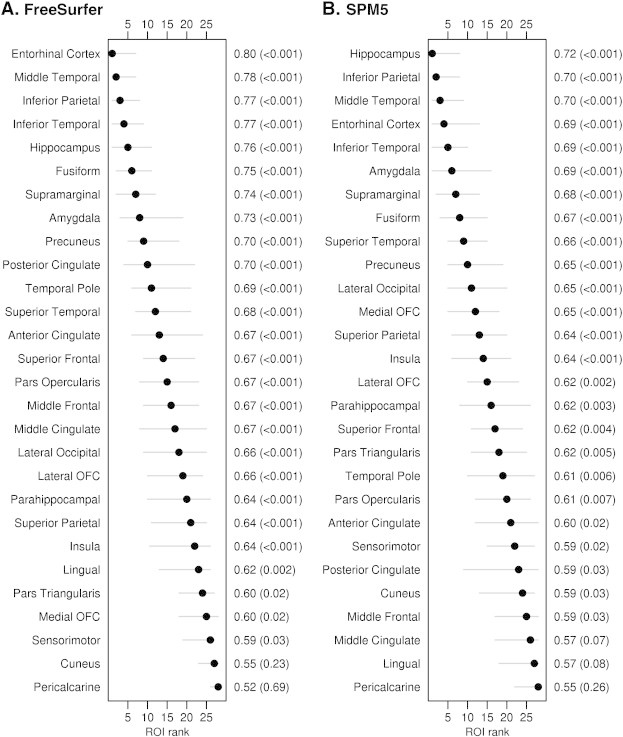
Forest plots illustrating ROI discrimination of CN − and MCI/AD + participants for FreeSurfer and SPM5. Regions-of-interest are ranked from best (rank of 1) to worst (rank of 28) according to AUROC and 95% confidence intervals are shown. The second y axis shows the AUROC values and p values.

**Fig. 3:**
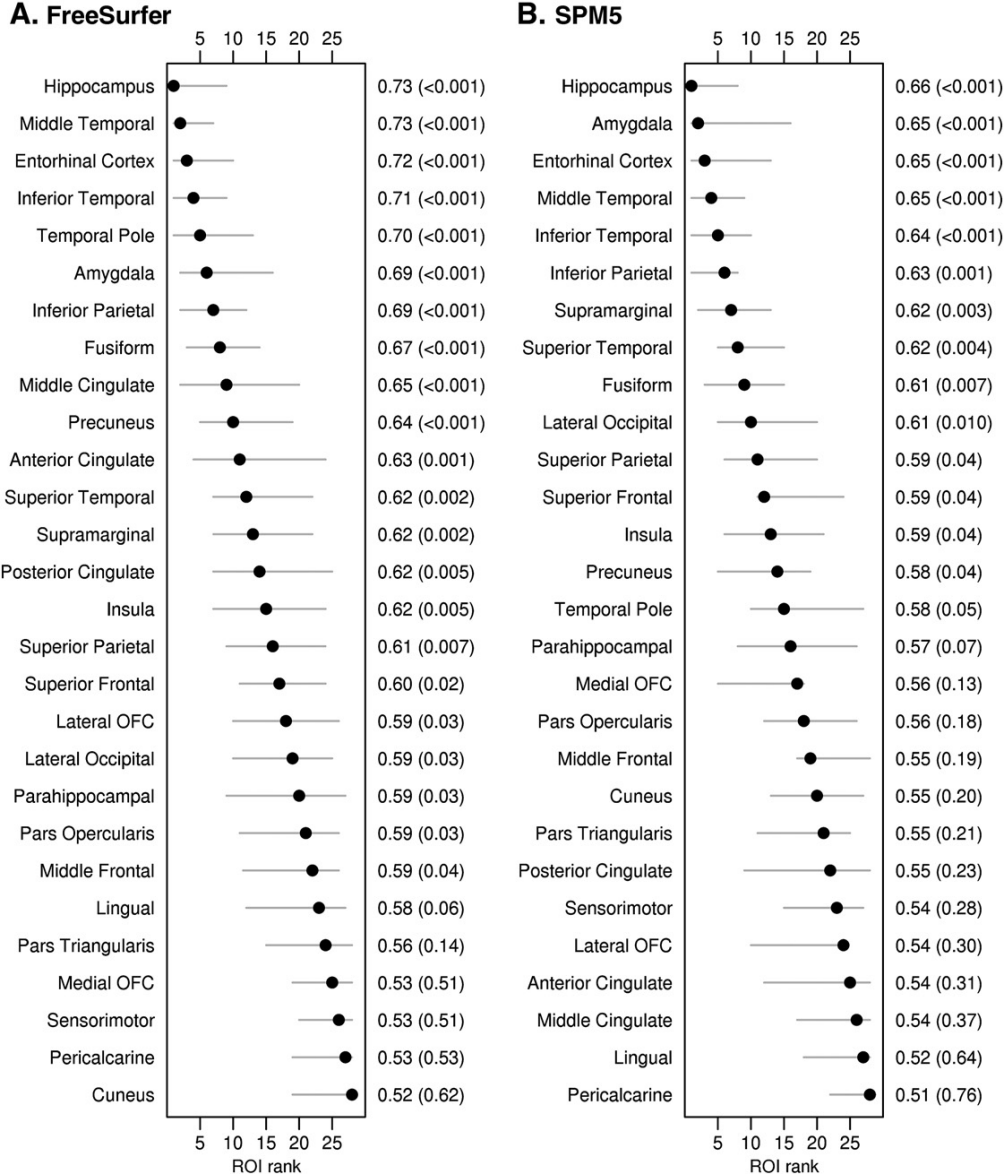
Forest plots illustrating ROI discrimination of CN + and MCI/AD + participants for FreeSurfer and SPM5. Regions-of-interest are ranked from best (rank of 1) to worst (rank of 28) according to AUROC and 95% confidence intervals are shown. The second y axis shows the AUROC values and p values.

In contrast, results from FreeSurfer and SPM5 were more consistent when comparing MCI/AD + to both CN − and CN + participants (Figs. 2 and 3). The entorhinal cortex, middle and inferior temporal gyrus, inferior parietal lobe, and hippocampus provided the best discrimination between CN − and MCI/AD +, with very similar regions identified in the comparisons of CN + and MCI/AD +. Confidence intervals for the rank of the ROIs were relatively narrow allowing meaningful inferences about where the greatest differences are to be found for both comparisons.

**Fig. 4:**
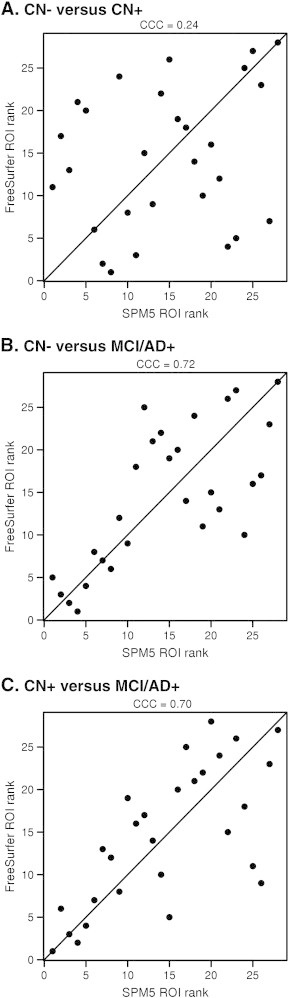
Plots illustrating concordance between the ranks of the 28 ROIs for FreeSurfer and SPM5. The degree of agreement is shown as a concordance correlation coefficient (CCC). Concordance across techniques was good for the CN − and CN + versus MCI/AD + comparisons, but poor for the CN − versus CN + comparison.

Overall, there was marginal discrimination ability and very poor agreement across FreeSurfer and SPM5 between ranks for discriminating CN − versus CN + (CCC = 0.24) but much better discrimination ability and agreement between the ranking for CN − versus MCI/AD + (CCC = 0.72) and CN + versus MCI/AD + (CCC = 0.70) (Fig. 4).

Poor agreement across technique for discriminating CN − versus CN + was also observed when utilizing a PiB cut-point of 1.4 (CCC = 0.25), removing patients with PiB values between 1.3 and 1.5 (CCC = 0.09), and when utilizing FreeSurfer volume measurements rather than thickness (CCC = 0.39). Excellent agreement was observed across technique in all the CN − versus MCI/AD + comparisons (CCC = 0.75, 0.75 and 0.80 respectively).

Correlations between the techniques varied considerably across ROIs. For all subjects, the median correlation across 28 ROIs was r = 0.31 (range 0.02–0.72). Correlations between FreeSurfer volume and SPM5 volume were similarly variable (median r = 0.45, range 0.20–0.72). Correlations between FreeSurfer thickness and FreeSurfer volume were somewhat higher on average (median r = 0.58, 0.40–0.78).

### 3.2 Whole-cortex/whole-brain analysis

**Fig. 5:**
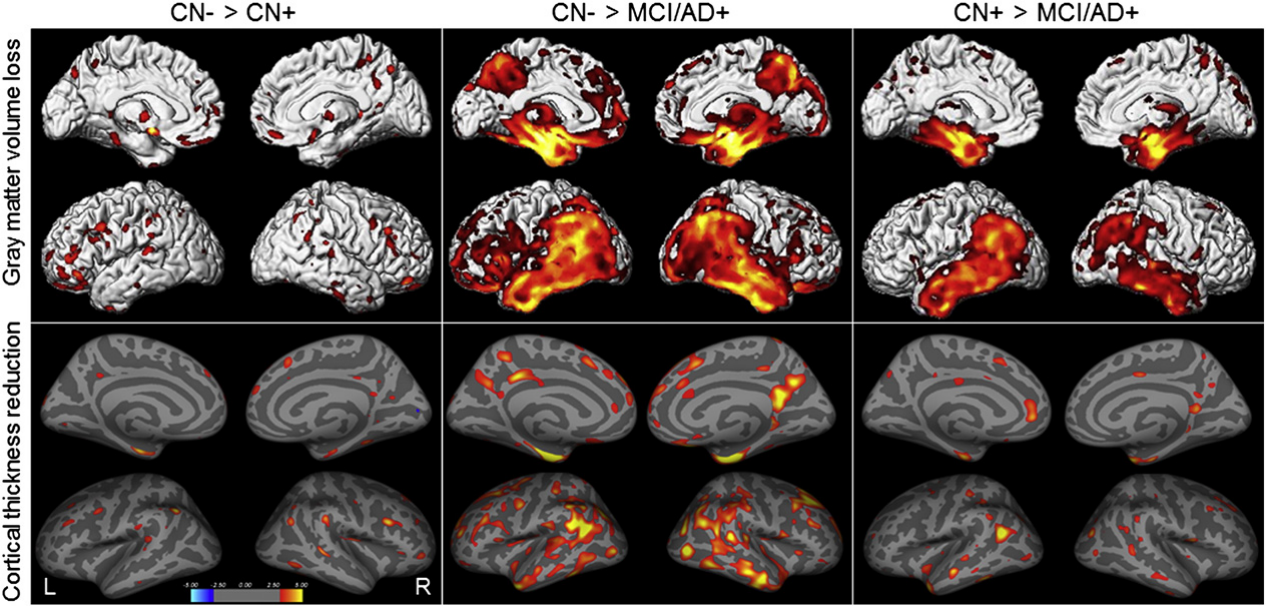
Voxel-level maps showing gray matter volume loss from SPM5 and cortical thickness reduction from FreeSurfer. Results are shown for comparisons between CN − and CN + participants, CN − and MCI/AD + participants, and CN + and MCI/AD + participants, uncorrected for multiple comparisons at p < 0.005.

Small scattered regions of significant differences were identified between CN − and CN + using both FreeSurfer and SPM5 at a very lenient statistical threshold of p < 0.005 (Fig. 5). The most significant peaks were identified in inferior and orbital frontal lobe in SPM5, and the entorhinal cortex, fusiform gyrus and lateral temporal, parietal and frontal lobes in FreeSurfer. The reverse comparison showed reduced thickness in pericalcarine cortex in FreeSurfer in CN − compared to CN +, but no regions that showed reduced volume in CN − compared to CN+ in SPM5. Both techniques showed abnormalities predominantly in medial temporal, lateral temporoparietal, and posteromedial cortices in MCI/AD + compared to CN − , with similar, although less significant, findings observed in the comparison to CN + (Fig. 5).

## 4 Discussion

The question of whether AD-related neurodegeneration can be detected in preclinical participants is critically important for the development of early imaging biomarkers. Two commonly used imaging techniques, FreeSurfer and SPM5, were utilized in this study to determine whether a consistent neuroanatomic signature could be identified in CN + participants.

Each technique identified significant regions of atrophy in CN + participants compared to CN − participants, explaining the higher proportion of subjective memory complaints in the CN + subjects. The patterns of cortical thinning in the CN + participants largely concurred with previous FreeSurfer studies of this population (Dickerson et al., 2009a, 2009b; Becker et al., 2011), and the patterns of gray matter volume loss were relatively consistent with another previous study that utilized SPM5, with both identifying relationships between PiB deposition and hippocampal atrophy (Chetelat et al., 2010); suggesting relatively consistent neuroanatomical signatures within technique. Despite these consistencies, however, we did not find consistent results across the two measurement techniques, both in the identification of most discriminatory ROIs and in the overall patterns of atrophy. One striking difference was that hippocampal volume was reduced in CN + participants using SPM5, but not FreeSurfer. The hippocampus is of particular interest because it is affected early in the disease in AD (Whitwell et al., 2007). It is one of the first structures to show tau deposition but is not heavily affected by Aβ deposition (Braak and Braak, 1991). The SPM5 results would suggest that hippocampal atrophy could potentially be a useful biomarker of preclinical AD in CN participants; likely reflecting the concurrent presence of hippocampal tau and neocortical Aβ deposition (Chetelat et al., 2010) in these participants. However, the FreeSurfer results suggest that hippocampal volume loss is not an early biomarker of AD, but that thinning of heteromodal association cortices are the earliest neuroanatomical changes, results that also replicate other prior findings (Sabuncu et al., 2011).

There are a couple of possible ways to interpret our findings. First, since the patterns of atrophy in CN + participants appear somewhat consistent within measurement technique across independent samples of participants, we could conclude that these are likely true biological effects. Differences observed across technique would then suggest that patterns of cortical thickness reduction and volume loss differ in these earliest stages of the disease, and that these two techniques are providing fundamentally different types of measurement of brain structure, as others have concluded (McKhann et al., 1984; Newcombe, 2006). However, concordance across FreeSurfer and SPM for the CN − versus CN + comparison was still poor when we assessed FreeSurfer volume rather than thickness, suggesting that our findings do not reflect fundamental differences between thickness and volume measures.

In addition, it is difficult to envision how measures of cortical thickness and cortical volume are not intimately related, measurement difficulties aside. Second, we could hypothesize that if any signature pattern of gray matter loss in CN + participants exists, the effects are subtle and easily overshadowed by measurement error. Differences across techniques could be driven by technical biases (Klauschen et al., 2009) and neither pattern may completely reflect a true biological effect. The high degree of variability between techniques and moderate correlations in hippocampal volume across techniques (r = 0.68 in CN − and r = 0.56 in CN +), despite being significant (p < 0.001), argues for this explanation. More generally, correlations between FreeSurfer thickness and SPM5 volume, FreeSurfer volume and SPM5 volume, or FreeSurfer thickness and FreeSurfer volume were moderate at best. Also arguing in favor of this latter explanation is the consistently weak discrimination ability of the “best” measures within each technique. The implications of this explanation would be that the presence of Aβ itself may not directly cause atrophy, as others have suggested (Josephs et al., 2008a; Dickerson et al., 2009b; Jack et al., 2010). It is also possible that one of the techniques is “correct” in the sense of being able to detect and localize differences in atrophy between CN + and CN −, and the other is incorrect. The accuracy of each technique may also vary regionally. There has been some suggestion that FreeSurfer is superior to SPM in detecting subtle cortical pathology (McKhann et al., 1984; Klauschen et al., 2009) and age-related gray matter loss (Hutton et al., 2009). The degree of variability and discriminatory ability between CN − and CN + was similar across techniques in this study however suggesting that this explanation is unlikely. However, there has been little direct comparison of the two techniques to assess strengths and weaknesses, particularly in the setting of subtle biological effects. The question of whether differences observed between FreeSurfer and SPM are biological or technical is still open, although this study demonstrates that evidence for a neuroanatomical signature of Aβ in CN participants is weak and hence that neuroanatomical patterns are unlikely to be useful biomarkers of preclinical AD at the individual participant level.

In contrast to the findings in CN + participants, the two techniques showed very consistent results in MCI/AD +. The severity of atrophy in MCI/AD + was greater when compared to CN − subjects, rather than CN + subjects, reflecting the greater regional variability observed in the CN + group; although in both comparisons the results were consistent across technique. Both techniques identified highly significant and similar patterns of loss involving regions that are typical for AD and are very similar to those identified in many previous studies using either image analysis technique (Du et al., 2007; Whitwell et al., 2007). Therefore, when the atrophic effects are larger there is considerable consistency across techniques in their detection. Not surprisingly given the larger effect sizes, confidence in the results was high for this comparison, with good agreement among ranks from the two techniques, less variability and excellent discrimination. This data supports the conclusion in the paragraph above that cortical thickness and volume are intimately related parameters. The distribution of atrophy identified in MCI/AD +, along with findings from previous imaging-pathology studies (Gosche et al., 2002; Jack et al., 2002; Josephs et al., 2008a; Whitwell et al., 2008), point towards an association between atrophy and the topographically concurrent deposition of tau.

The strengths of our study include the fact that we had a large cohort of well-matched CN participants, and that we utilized two different but widely used and thoroughly validated image analysis techniques which allowed us to assess consistency in the findings. Importantly, we also demonstrate that our results were robust to varying the PiB cut-point. The two techniques use different approaches to assess cortical gray matter. Further, the atlases used to parcellate the brain differed across techniques which meant that while we created a common set of 28 ROIs for each method, the boundaries of each ROI may not have been identical. The templates used for normalization also differed across technique although not dramatically. These technical differences may contribute to the relatively poor cross-technique correspondence. In addition, the averaging of left and right hemisphere volumes and thicknesses may have reduced sensitivity. Nevertheless, the consistent findings across technique in MCI/AD + suggest that the two methods are comparable when a larger disease effect is present. It will be crucial for future studies to focus on technical comparisons, particularly to identify the optimal technique for detecting the earliest signs of atrophy in particular brain regions. The presence of subjective memory complaints has also been suggested to influence the relationship between amyloid deposition and atrophy (Chetelat et al., 2010), and will require further investigation. Another important limitation of the study was that while we had a measure of Aβ, we did not have a measure of tau in these CN participants, so it is impossible to determine whether participants with evidence of fibrillar brain amyloid also have evidence of substantial tau pathology. Other proteins are also often present in the brains of AD participants that could influence the patterns of atrophy, such as TDP-43 (Josephs et al., 2008b).

The weak and inconsistent nature of the findings across technique in this study cast doubt on the existence of a neuroanatomical signature of preclinical AD in PiB-positive CN participants, and do not support the suggestion that Aβ deposition is related to spatially coincident neurodegeneration. The topography of the SPM5 results suggests a closer relationship between atrophy and tau pathology. However, definitive resolution will need to await the availability of a fully validated tau PET ligand (Fodero-Tavoletti et al., 2011).

The following are the supplementary data related to this article.

Supplemental Table 1 Key to describe how ROIs from both FreeSurfer and the SPM5 AAL atlas were combined to create the 28 master ROIs used in this study.

Supplementary data to this article can be found online at http://dx.doi.org/10.1016/j.nicl.2013.01.006.

## Role of the funding source

This study was supported by NIH grants P50-AG16574, U01-AG06786, and R01-AG11378. The funding organizations played no role in the conduct of the study; collection, management, analysis, or interpretation of the data; or preparation, review, or approval of the manuscript.

## Disclosure statement

The authors have no conflicts of interest.

## Figures and Tables

**Table 1:** Participant demographics.

	CN − (n = 115)	CN + (n = 115)	CN − versus CN + P-value	MCI/AD + (n = 88)	CN − versus MCI/AD + P-value
No. of females (%)	52 (45)	49 (43)	0.69	41 (47)	0.85
Age, years	80 (71, 92)	80 (60, 93)	0.87	79 (51, 94)	0.34
Education, years	14 (11, 20)	15 (8, 20)	0.46	14 (7, 20)	0.85
Short Test of Mental Status score	35 (27, 38)	35 (29, 38)	0.20	30 (4, 36)^a^	< 0.001
CDR Sum of Boxes	0.0 (0, 1.5)	0.0 (0, 1.5)	0.19	1.5 (0, 13.0)	< 0.001
No. *APOE* ε4 positive (%)^b^	19 (17)	41 (38)	< 0.001	51 (65)	< 0.001
Subjective memory complaints (%)^c^	83 (73%)	97 (86%)	0.03	–	–

Note: Unless otherwise indicated, values shown are median (range). Abbreviations: CN −, cognitively normal and negative for PiB (defined as < 1.5); CN +, cognitively normal and positive for PiB (defined as > 1.5); MCI/AD +, mild cognitive impairment or Alzheimer's disease and positive for PiB (defined as > 1.5); CDR, Clinical Dementia Rating scale; *APOE*, Apolipoprotein E.

a Converted from the Mini Mental State Exam for some participants.

b Twenty-one patients had unknown *APOE* status (3 in CN −, 8 in CN +, and 10 in MCI/AD +).

c Data available in 113 CN − and 113 CN + subjects.
