# Supplementary material for: Does amyloid deposition produce a specific atrophic signature in cognitively normal subjects?
Source: Neuroimage Clin. 2013 Jan 24;2:249–57. doi: 10.1016/j.nicl.2013.01.006 (PMC3778266; doi:10.1016/j.nicl.2013.01.006)
Supplement: Supplemental Table 1 — Key to describe how ROIs from both FreeSurfer and the SPM5 AAL atlas were combined to create the 28 master ROIs used in this study. [file mmc1.doc]

**Supplemental Table 1: Key to describe how ROIs from both FreeSurfer and the SPM5 AAL atlas were combined to create the 28 master ROIs used in this study.**

| **New regions used in study** | **FreeSurfer regions** | **SPM5 regions** |
| --- | --- | --- |
| Superior frontal | Superior frontal | Frontal sup + Frontal sup medial + Supp motor area |
| Middle frontal | Rostral middle frontal + Caudal middle frontal | Frontal mid |
| Pars opercularis | Pars opercularis | Frontal inf oper |
| Pars triangularis | Pars triangularis | Frontal inf tri |
| Lateral orbitofrontal | Lateral orbitofrontal + Pars orbitalis | Frontal sup orb + Frontal mid orb + Frontal inf orb |
| Medial orbitofrontal | Medial orbito frontal + Frontal pole | Frontal med orb + Rectus + Olfactory |
| Superior parietal | Superior parietal | Parietal sup |
| Inferior parietal | Inferior parietal | Parietal inferior + Angular |
| Supramarginal | Supramarginal | Supramarginal |
| Inferior temporal | Inferior temporal | Temporal inf |
| Middle temporal | Middle temporal | Temporal mid |
| Superior temporal | Superior temporal + bankssts +  Transverse Temporal | Temporal sup + Temporal pole sub +  Heschl |
| Fusiform | Fusiform | Fusiform |
| Temporal pole | Temporal pole | Temporal pole mid |
| Parahippocampal | Parahippocampal | Parahippocampal |
| Entorhinal | Entorhinal | Entorhinal cortex |
| Hippocampus | Hippocampus | Hippocampus |
| Amygdala | Amygdala | Amygdala |
| Lateral occipital | Lateral occipital | Occipital sup + Occipital mid + Occipital inf |
| Lingual | Lingual | Lingual |
| Cuneus | Cuneus | Cuneus |
| Pericalcarine | Pericalcarine | Calcarine |
| Precuneus | Precuneus | Precuneus |
| Anterior cingulate | Caudal anterior cingulate + Rostral anterior cingulate | Cingulum ant |
| Middle cingulate | Posterior cingulate | Cingulum mid |
| Posterior Cingulate | Isthmus cingulate | Cingulum post |
| Insula | Insula | Insula |
| Sensorimotor | Postcentral + Precentral + Paracentral | Postcentral +  Precentral + Paracentral lobule + Rolandic oper |
